# Energy Expenditure, Body Composition, and Skeletal Muscle Oxidative Capacity in Patients with Myotonic Dystrophy Type 1

**DOI:** 10.3233/JND-230036

**Published:** 2023-07-04

**Authors:** Isis B.T. Joosten, Cas J. Fuchs, Milou Beelen, Guy Plasqui, Luc J.C. van Loon, Catharina G. Faber

**Affiliations:** a Department of Neurology and MHeNS School for Mental Health and Neuroscience, Maastricht University Medical Centre+, Maastricht, The Netherlands; b Department of Human Biology, NUTRIM School of Nutrition and Translational Research in Metabolism, Maastricht University Medical Centre+, Maastricht, The Netherlands; c Department of Physical Therapy, Maastricht University Medical Centre+, Maastricht, The Netherlands; d Department of Nutrition and Movement Sciences, NUTRIM School of Nutrition and Translational Research in Metabolism, Maastricht University Medical Centre+, Maastricht, The Netherlands

**Keywords:** Myotonic dystrophy, energy expenditure, body composition, physical activity

## Abstract

**Background:**

Myotonic dystrophy type 1 (DM1) patients are at risk for metabolic abnormalities and commonly experience overweight and obesity. Possibly, weight issues result from lowered resting energy expenditure (EE) and impaired muscle oxidative metabolism.

**Objectives:**

This study aims to assess EE, body composition, and muscle oxidative capacity in patients with DM1 compared to age-, sex- and BMI-matched controls.

**Methods:**

A prospective case control study was conducted including 15 DM1 patients and 15 matched controls. Participants underwent state-of-the-art methodologies including 24 h whole room calorimetry, doubly labeled water and accelerometer analysis under 15-days of free-living conditions, muscle biopsy, full body magnetic resonance imaging (MRI), dual-energy x-ray absorptiometry (DEXA), computed tomography (CT) upper leg, and cardiopulmonary exercise testing.

**Results:**

Fat ratio determined by full body MRI was significantly higher in DM1 patients (56 [49–62] %) compared to healthy controls (44 [37–52] % ; *p* = 0.027). Resting EE did not differ between groups (1948 [1742–2146] vs (2001 [1853–2425>] kcal/24 h, respectively; *p* = 0.466). In contrast, total EE was 23% lower in DM1 patients (2162 [1794–2494] vs 2814 [2424–3310] kcal/24 h; *p* = 0.027). Also, DM1 patients had 63% less steps (3090 [2263–5063] vs 8283 [6855–11485] steps/24 h; *p* = 0.003) and a significantly lower VO_2_ peak (22 [17–24] vs 33 [26–39] mL/min/kg; *p* = 0.003) compared to the healthy controls. Muscle biopsy citrate synthase activity did not differ between groups (15.4 [13.3–20.0] vs 20.1 [16.6–25.8] μM/g/min, respectively; *p* = 0.449).

**Conclusions:**

Resting EE does not differ between DM1 patients and healthy, matched controls when assessed under standardized circumstances. However, under free living conditions, total EE is substantially reduced in DM1 patients due to a lower physical activity level. The sedentary lifestyle of DM1 patients seems responsible for the undesirable changes in body composition and aerobic capacity.

## INTRODUCTION

Myotonic dystrophy type 1 (DM1) is an autosomal dominantly inherited disease characterized by its progressive multisystemic nature. While muscle weakness and myotonia (inability to relax muscles) form the basis of the DM1 phenotype, organ involvement can cause possible life-threatening complications during the course of disease. Since curative or disease modifying treatment options are currently unavailable, disease management focusses on early detection of organ involvement and improving quality of life [1].

The genetic basis of DM1 lies in a CTG repeat expansion located on chromosome 19, with repeat lengths being roughly correlated with disease severity [2, 3]. Based on symptomatology, age of onset and CTG repeat length, DM1 is often classified into four clinical subtypes [2, 4]. The adult-onset subtype is the most prevalent and has been linked to profound organ involvement [2].

Apart from organ complications, DM1 is known to have metabolic consequences such as insulin resistance, and increased levels of total cholesterol, low-density lipoprotein and triglycerides imposing possible risk factors for cardiovascular disease [5]. Overweight and obesity are also frequently observed [6–9], with a prevalence of 28 and 22%, respectively, in our own DM1 population of 493 patients (*unpublished data*). Moreover, alterations in body composition in this patient population have been described previously [6, 10].

Overweight, obesity and other adverse changes in body composition in DM1 may result from decreased physical activity due to motor impairments and/or from the lack of motivation due to neuropsychological expression of disease. However, literature also suggests that resting energy expenditure (EE) may be lower in DM1 patients and that muscle oxidative metabolism could be impaired [11, 12]. To accurately assess potential derangements in EE in these patients, differences in body composition and habitual physical activity need to be taken into account. In this study we applied various state-of-the-art methodologies to assess EE, body composition, and skeletal muscle oxidative capacity in 15 patients with DM1 as well as 15 healthy age-, sex- and BMI-matched controls.

## MATERIALS AND METHODS

### Study design and participants

A prospective cross-sectional case control study was conducted including 15 DM1 patients and 15 healthy age-, sex- and BMI-matched controls. Age matching was based on decades (18–29, 30–39, 40–49, 50–59, 60–69 and >70 years). BMI matching was based on the WHO BMI classification [13]: BMI 18.5–24.9, 25–29.9, 30–35 kg/m^2^. Each healthy volunteer originated from the same age- and BMI group as the matched DM1 patient, and was of the same sex. In order to be eligible to participate, subjects had to be 18 years or older. DM1 patients had to have a genetically confirmed DM1 diagnosis of the adult-onset subtype, had to be able to walk and cycle, and have a muscular impairment rating scale (MIRS, a DM1 specific scale for muscle weakness) score <5 [14]. Exclusion criteria consisted of: use of medication interacting with muscle metabolism (such as steroids and statins), diabetes mellitus, weight loss of more than 3 kg within the last three months, and the use of protein supplements. Healthy volunteers were also excluded in case of a diagnosed neuromuscular disorder, an abnormal neurological examination, or another medical condition possibly interfering with muscle strength or muscle mass. Patients and healthy volunteers were recruited between 2019–2021 from the neurology outpatient clinic of the Maastricht University Medical Centre+ and through the Dutch DM1 expertise centre website (mdexpertisecentrum.nl). As similar studies have not been conducted in this specific or comparable patient populations, we were unable to determine an estimated effect size for standard sample size calculation. Literature suggests that sample sizes for pilot studies must include a minimum of 12 participants per group [15]. When taking possible drop-out rates of 10–20% into account, an amount of 15 participants per group was established.

### Standard protocol approvals, registrations, and patient consents

The study was conducted in accordance with the Declaration of Helsinki, and the research protocol was approved by the institutional Medical Ethics Committee (METC 182009, approved on 27-06-2018). Written informed consent was obtained from all included participants.

### Study procedures

**Fig. 1:**
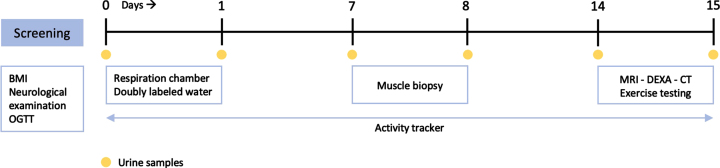
Overview of 15-day study period. BMI, body mass index; CT, computed tomography; DEXA, dual-energy X-ray absorptiometry; MRI, magnetic resonance imaging, OGTT, oral glucose tolerance testing.

Eligible participants were invited for screening, to verify in- and exclusion criteria and to confirm adequate matching. At screening, medical history, medication use, and body weight and height were determined. Fasting glucose was assessed by finger-prick glucose test, with a fasting glucose of >7 mmol/L being considered an exclusion criterion. Neurological examination was performed by a trained neuromuscular neurologist, to determine the degree of muscle weakness and MIRS score in patients, and to confirm normal strength in healthy participants. A trained neuromuscular neurologist evaluated subjects’ eligibility based on in- and exclusion criteria and matching criteria. In case of confirmed eligibility, a 2-hour 7-point oral glucose tolerance test (OGTT) was performed on the day of screening. Thereafter, participants completed a 15-day study period (Fig. 1):

• Day 0-1: 24-hour resting EE was determined by whole room calorimetry (see **Respiration Chamber**). Baseline urine samples were collected for doubly labeled water (DLW) analysis of total EE under free living conditions (see **Doubly Labeled Water**). DLW bolus was then administered orally to participants in the controlled environment of the respiration chamber (RC). Another urine sample was collected upon exiting the RC. An activity monitor and corresponding instructions were provided upon leaving the RC after 24-hours, and participants were asked to wear their monitor daily until study day 15 (see **Activity Monitor**).
• Day 1–14: Urine was collected at home on the evening of day 7 and morning of day 8. On day 8, participants visited the hospital to hand in urine samples and undergo muscle biopsy (see **Muscle Biopsy**). After leaving the hospital on day 8, participants returned to free living conditions while still wearing the activity monitor. Another urine sample was collected for DLW analysis on the evening of day 14.
• Day 15: On the morning of day 15, participants collected a urine sample at home and returned to the hospital. In the hospital, subjects handed in urine samples and underwent a full body magnetic resonance imaging (MRI), dual-energy x-ray absorptiometry (DEXA) and single slice computed tomography (CT) scan of the upper leg to assess body composition and muscle mass (see **Full Body MRI, CT upper leg** and **DEXA)**. Exercise testing for determination of maximum oxygen uptake capacity was performed (see **Exercise testing**).

### Oral glucose tolerance test

Glucose tolerance was determined by a standardized 7-point OGTT that was performed after an overnight fast, using a 200 mL glucose solution containing 75 grams of glucose. Blood samples were collected at *t* = 0, 10, 20, 30, 60, 90 and 120 minutes for the measurement of plasma glucose concentrations. OGTT was considered normal in case of a fasting plasma glucose <5.6 mmol/L and a plasma glucose <7.8 mmol/L at *t* = 120 minutes. Impaired fasting glucose was defined as a fasting plasma glucose of 5.6–6.9 mmol/L [16]. Impaired glucose tolerance was defined as a plasma glucose of 7.8–11.0 mmol/L at *t* = 120 minutes [16].

### Respiration chamber

Subjects resided in a RC for whole room calorimetry from 09:00 PM on day 0 until 09:00 PM on day 1. The RCs are located at the Metabolic Research Unit Maastricht (Maastricht University). 24-hour resting EE was determined through continuous monitoring and analysis of O_2_ and CO_2_ concentrations of incoming and outgoing air. O_2_ consumption and CO_2_ production rates were expressed in mL per min, measured at 5 min intervals over a 24-hour period. 24-hour resting EE was calculated using mean O_2_ consumption and mean CO_2_ production rates in mL/min in Weir’s formula [17]. Subjects consumed standardized meals (16% protein, 55% carbohydrate, 28% fat) and were instructed to avoid exhaustive physical activity during the 24-hour stay. Participants did not receive instructions on (hours of) sleep, but were served breakfast at a standardized time.

### Doubly labeled water

DLW bolus consisted of a small volume of water (80–160 mL depending on body weight) containing 5 Atomic percent (At%) ^2^H_2_O and 10 At% H_2_^18^O per os, resulting in an initial excess body water enrichment of 150 ppm for ^2^H and 300 ppm for ^18^O. Urine samples were collected at timepoints described above and stored in a cooled environment, until final storage within 12 hours after collection in an air-tight screw-capped glass container, in a –30°C freezer. At the end of the study, urine samples were analysed according to The Maastricht Protocol [18]. Excess disappearance rate of ^18^O relative to ^2^H through urine was converted to an estimate of total EE according to the Maastricht protocol for the measurement of body composition and EE with labeled water [18–20].

### Activity monitor

Participants wore a Philips Actical accelerometer attached to a belt on the left hip during a 14-day period, starting directly after leaving the RC until study day 15. Participants were instructed to wear the accelerometer at all times, except for when sleeping or showering. Accelerations were converted into number of steps per 24 hours and % time sedentary per 24 hours by Actical software. We used data of 12 full measuring days for analysis as participants resided in the hospital on study day 8 and 15 and had to take off the accelerometer during and between measurements.

### Muscle biopsy

A muscle biopsy was obtained 15 cm above the patella from the vastus lateralis muscle approximately 2 cm below the fascia, by percutaneous needle biopsy technique as described by Bergström et al. [21]. Skin and muscle fascia were locally anesthetized using 1% xylocaine. Mitochondrial citrate synthase (CS) activity was measured as previously described [22, 23].

### Full body MRI

Full body MRI was performed using a 3T MAGNETOM Prisma Fit scanner (Siemens Healthcare, Erlangen, Germany) using the whole-body coil and the body array coil 18 elements (Siemens Healthcare, Erlangen, Germany). A 6-minute dual-echo Dixon Vibe protocol was applied, providing a water and fat separated volumetric data set covering head to ankles. In total 8 slabs were acquired containing all individual images. Common scanning parameters for all included slabs were: flip angle (α) = 10°, repetition time (TR) = 3.89 milliseconds, echo time (TE) = 1.22/2.45 milliseconds, bandwidth = 930 Hz/Px and 256×192 matrix. There was no interslice gap (0 mm). Slabs covering the abdomen were acquired during 18-second expiration breath-holds and consisted of 44 slices with a voxel size 2.0×2.0×5.0 mm^3^. The slabs covering the legs, consisted of 88 slices with a voxel size of 2.0×2.0×3.0 mm3. Body composition analysis was performed by using the AMRA Profiler Research (AMRA Medical AB, Linköping, Sweden) [24–26]. Scans were analyzed for total lean tissue volume (including thigh muscle volume), total adipose tissue volume (including visceral adipose tissue volume and subcutaneous adipose tissue volume), posterior thigh muscle fat infiltration (expressed in %) and fat ratio (expressed in %). Fat ratio was defined as: (visceral adipose tissue volume + subcutaneous adipose tissue volume) / (visceral adipose tissue volume + subcutaneous adipose tissue volume + thigh muscle volume). Following the automated segmentation and analysis process, an experienced operator reviewed each segmentation for anatomical correctness and technical quality.

### CT upper leg

Muscle cross-sectional area (as a proxy for muscle mass) was assessed from a single-slice CT scan (Somatom Definition Flash; Siemens Healthineers, Forchheim, Germany). While subjects were lying supine, with their legs extended and their feet secured, a 2-mm thick axial image was taken 15 cm proximal to the top of the patella. Analysis included manual tracing of the quadriceps muscle using ImageJ software by an experienced and blinded researcher, to determine cross-sectional area (CSA) of the quadriceps, as previously described [27].

### Dual-energy X ray Absorptiometry (DEXA)

A DEXA scan was performed on a commercially available clinical system (Discovery A; Hologic, Bedford, MA, USA) with system’s software package (Hologic-Apex software, version 4.5.3, with viewer software Hologic Physician’s viewer, version 7.1) to determine total lean mass and fat mass.

### Exercise testing

Aerobic capacity was tested with a cardiopulmonary exercise test to exhaustion with continuous electrocardiography and respiratory gas analysis. Tests were performed on a cycle ergometer (Lode Corival, Groningen, the Netherlands). Ventilatory parameters were measured breath-by-breath (Carefusion; San Diego, USA). After 3 min of unloaded cycling, the workload was increased according to an individualized ramp protocol aiming at a total test duration of 8–12 minutes. Subjects were instructed to cycle with a pedaling rate of >60 revolutions min^–1^. The test was ended when the subjects stopped cycling or were unable to maintain the required pedaling frequency. Maximal workload (Wmax), peak oxygen uptake (VO_2_peak) and peak respiratory exchange ratio (RER_peak_) were recorded as the final 30 sec averaged value of the test.

### DNA analysis

DNA analysis took place at DM1 diagnosis, as standard of care. CTG-repeat lengths were determined by analyzing DNA extracted from peripheral blood samples through polymerase chain reaction (PCR), followed by fragment length analysis and Southern blot analysis.

### Statistical analysis

Statistical analysis was performed using IBM SPSS version 25 (SPSS Inc, Chicago, IL). The distribution of continuous variables was assessed for normality, by visual inspection of histograms and standardized normal probability plots. Continuous variables are expressed as mean±standard deviation (SD) or as median with interquartile range (IQR) in case of skewness. Categorical variables are expressed as counts (percentages). Differences between groups were compared using the chi-squared (χ^2^) test or Fisher’s exact test (categorical data), and the unpaired Student’s *t*-test or the Mann-Whitney U test (continuous variables). *P* values of <0.05 were considered statistically significant.

## RESULTS

### Study population

Fifteen patients diagnosed with DM1 and 15 healthy matched controls were included in the study (Table 1). CTG repeat length and MIRS classification of DM1 patients are described in Table 1. Fasting blood glucose concentrations were normal in all participants, except for 6 DM1 patients who met the criteria for having an impaired glucose tolerance based on abnormal OGTT values. Missing data consisted of one full body MRI in the DM1 group, one CT upper leg in the DM1 group and one muscle biopsy in the healthy control group.

**Table 1:** Baseline characteristics

	Myotonic dystrophy	Healthy controls	*P* value
	patients (*n* = 15)	(*n* = 15)
Age, median [IQR]	41 [30–50]	42 [30–50]	1.000
Male, *n* (%)	7 (47%)	7 (47%)	1.000
BMI, median [IQR]	26.5 [21.8–28.6]	26.2 [22.9–29.4]	1.000
BMI category, *n* (%)
18.5–24.9 kg/m^2^	6 (40%)	6 (40%)	1.000
25–29.9 kg/m^2^	7 (47%)	7 (47%)	1.000
30–35 kg/m^2^	2 (13%)	2 (13%)	1.000
CTG repeat length, *n* (%)		
100–150	3 (20%)	
>150	6 (40%)	
>200	4 (27%)	
>350	2 (13%)	
MIRS, *n* (%)		
1	0 (0%)	
2	2 (13%)	
3	5 (33%)	
4	8 (54%)	
Patients with abnormal fasting glucose, *n* (%)	0 (0%)	0 (0%)	1.000
Patients with impaired glucose tolerance, *n* (%)	6 (40%)	0 (0%)	0.006

BMI, body mass index; MIRS, muscular impairment rating scale.

### Body composition

**Fig. 2:**
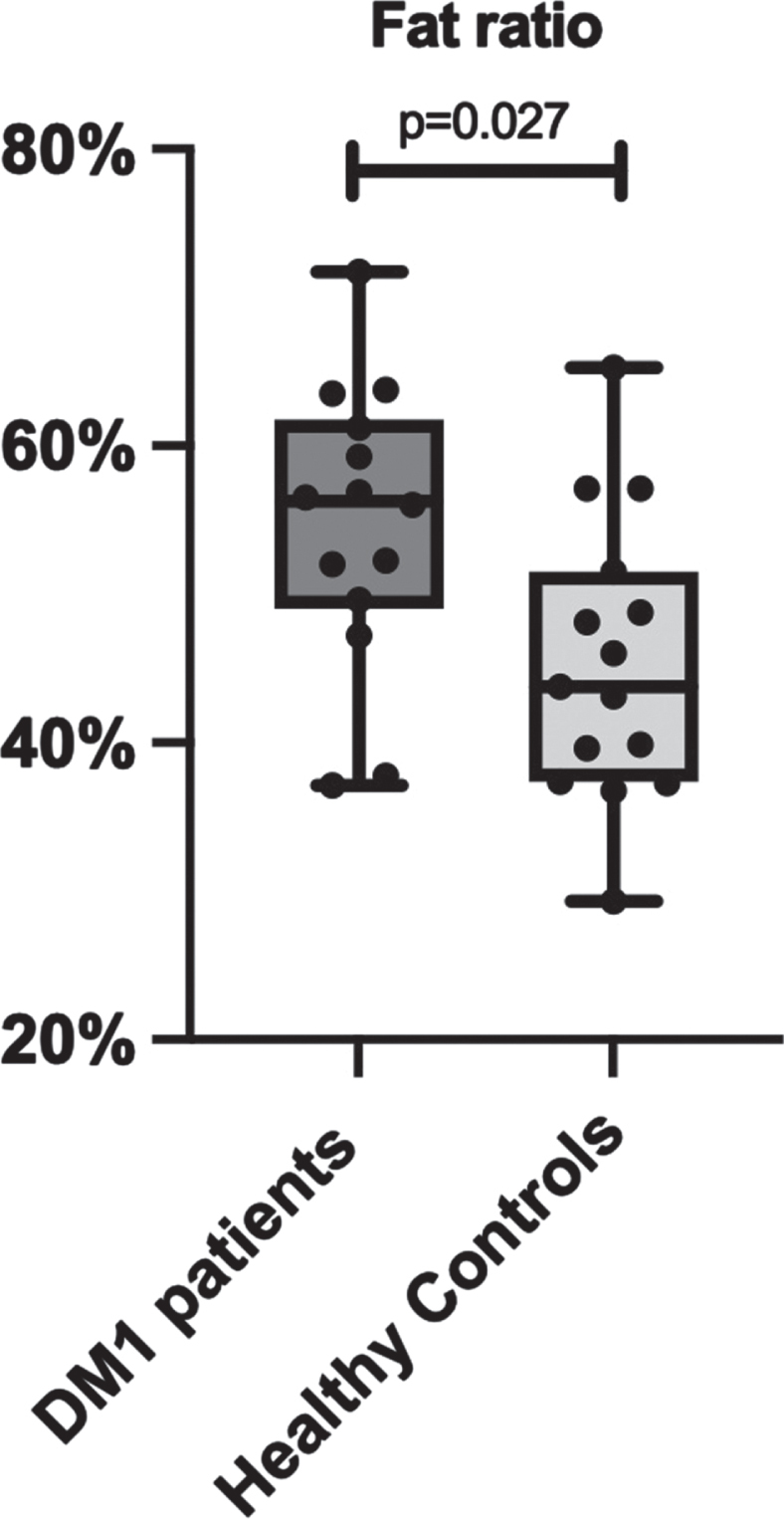
Comparison of fat ratio between myotonic dystrophy type 1 (DM1) patients and healthy age-, sex- and BMI-matched controls, based on full-body MRI measurements.

**Fig. 3:**
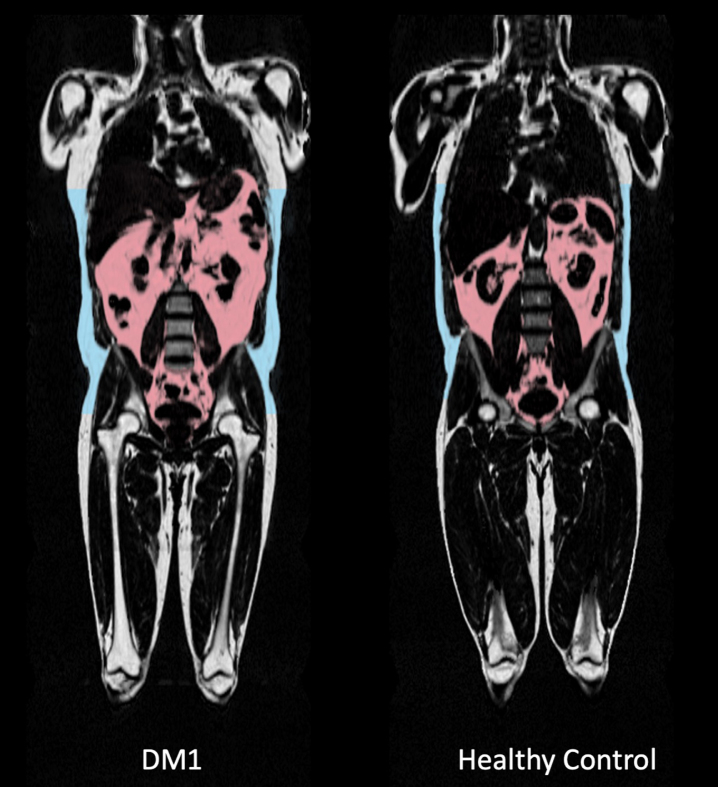
Comparison of full body MRI images between a myotonic dystrophy type 1 (DM1) patient and a healthy age-, sex- and BMI-matched control. Visceral adipose tissue (VAT) deposition is highlighted in pink. VAT volume was 5,59L in the displayed DM1 patient versus 4,28 L in the healthy matched control. Subcutaneous fat deposition is highlighted in blue, as part of the total adipose tissue volume. Also note the difference in shoulder and leg musculature: thigh lean muscle volume was 11,1 L in the DM1 patient versus 15,1 L in the healthy control, as part of total lean tissue volume.

Total lean and adipose tissue volumes determined by full-body MRI were not statistically different between groups, with median total lean tissue volume 19.81 [18.81–23.19] L in the DM1 group versus 22.74 [20.54–28.72] L in the control group (*p* = 0.066). Median total adipose tissue volume was 25.11 [19.31–31.33] L in DM1 patients versus 20.82 [16.65–28.25] L in healthy controls (*p* = 0.466). Fat ratio was significantly higher in DM1 affected individuals (median 56 [49–62] %) than in healthy controls (median 44 [37–52] % ; *p* = 0.027; Fig. 2). Also, visceral adipose tissue volume was significantly higher in DM1 patients when compared with healthy controls (median 5.06 [2.66–6.06] vs 2.79 [1.51–4.28] L, *p* = 0.027). A representative image of the adipose tissue assessment for both a DM1 patient and a healthy control is given in Fig. 3. Furthermore, DM1 patients had significantly more posterior thigh muscle fat infiltration than healthy adults (median muscle fat infiltration 12.43 [9.07–13.99] % in DM1 patients versus 9.62 [8.71–11.80] % in healthy adults; *p* = 0.027). Body composition was also assessed by using DEXA scan. Total fat and total lean mass determined by DEXA were not statistically different between groups (see Supplementary Table 1). In addition, CT assessment of quadriceps CSA tended to be lower in the DM1 patients, with a CSA of 5533 [5287–6431] mm^2^ in DM1 patients vs 7057 [5965–7881] mm^2^ in healthy controls (*p* = 0.066).

### Energy expenditure

**Fig. 4:**
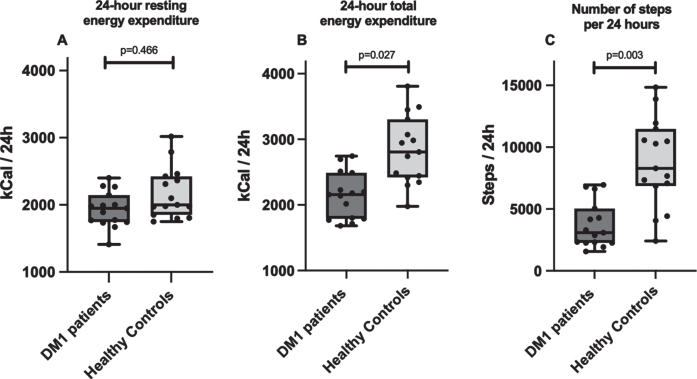
Comparison between A) 24 h resting energy expenditure measured by whole room calorimetry, B) 24 h total energy expenditure under free living conditions measured using doubly labeled water, and C) number of steps per 24 h measured by accelerometer, in myotonic dystrophy type 1 patients and healthy age-, sex- and BMI-matched controls. DM1, myotonic dystrophy type 1.

Median 24-hour resting EE determined by RC did not differ between DM1 patients (1948 [1742–2146] kcal/24 h) and healthy controls (2001 [1853–2425] kcal/24 h; *p* = 0.466; Fig. 4A). After correction for lean tissue volume, resting EE per liter lean tissue was 89 [86–100] kcal/L in DM1 patients and 90 [84–97] kcal/L in matched controls (*p* = 0.715). In contrast, total EE assessed under free living conditions via DLW measurement was 23% lower in DM1 affected individuals (2162 [1794–2494] kcal/24 h) when compared with healthy controls (2814 [2424–3310] kcal/24 h; *p* = 0.027; Fig. 4B).

### Physical activity

There was a significant difference in the amount of habitual daily physical activity performed between both groups. DM1 patients had a 63% lower amount of steps per 24 hours, with a median number of 3090 [2263–5063] steps/24 h vs 8283 [6855–11485] steps/24 h in healthy adults (*p* = 0.003, Fig. 4C). Individuals in the DM1 group were sedentary 86 [82–91] % of the time, in comparison to 79 [75–84] % in the matched control group (*p* = 0.027).

### Muscle oxidative capacity

DM1 patients had a significantly lower median VO_2_ peak of 22 [17–24] mL/min/kg in comparison to 33 [26–39] mL/min/kg in healthy matched adults (*p* = 0.003). Median VO_2_ peak stratified according to sex is displayed in Table 2. There was a statistically significant difference in the absolute values at which the ventilatory anaerobic threshold and respiratory compensation point were reached (Table 2). These differences were no longer significant when expressed as percentages of the VO_2_ peak (Table 2). Also, there was a statistically significant difference in maximal workload and oxygen uptake efficiency slope (Table 2). Median respiratory exchange ratio did not differ between groups (Table 2).

**Table 2:** Exercise testing results

	DM1 patients	Healthy controls	*P* value
	*n* = 15	*n* = 15
VO_2_ peak, mL/min/kg	22 [17–24]	33 [26–39]	0.003
Males	23 [19–33]	39 [30–41]
Females	21 [14–23]	31 [25–37]
Ventilatory anaerobic threshold, mL/min/kg	15 [11–17]	20 [16–22]	0.011
% of VO_2_ peak	57% [53–60]	60% [53–62]	1.000
Respiratory compensation point, mL/min/kg	22 [18–28]	31 [24–34]	0.019
% of VO_2_ peak	91% [84–95]	89% [86–95]	0.414
Respiratory exchange ratio	1.22 [1.06–1.28]	1.18 [1.12–1.11]	1.000
Maximal workload, W	139 [111–160]	217 [191–275]	0.003
Oxygen Uptake Efficiency Slope, ([mL/min O_2_]/[L/min VE])	1851 [1689–2201]	2711 [2298–3306]	0.003

DM1, myotonic dystrophy type 1.

### Muscle biopsy

CS activity in muscle biopsy material did not differ significantly between groups, with a median of 15.4 [13.3–20.0] μM/g/min in DM1 patients versus 20.1 [16.6–25.8] μM/g/min in healthy adults (*p* = 0.449).

## DISCUSSION

In the present study we observed a higher fat ratio in patients affected by DM1 when compared to healthy, matched controls. The difference in body composition was accompanied by a 23% lower total EE in DM1 patients when compared to healthy matched controls, assessed under free living conditions. In line, habitual physical activity level was 63% lower in patients compared to healthy controls, with substantially more time spent in a sedentary state. The lower EE could not be attributed to any metabolic derangements as 24 h EE under standardized conditions did not differ between groups. Aerobic capacity, assessed by oxygen uptake capacity, was substantially reduced in DM1 patients when compared to sex- and age-matched controls.

Apart from overweight in more than 50% of the general DM1 population [6–9], body composition has been described to be altered with increased fat mass, decreased lean body mass and visceral obesity [6, 7, 10, 28, 29]. In line with previous studies [6, 7, 10, 28], we performed DEXA to assess body composition. We observed that DEXA-derived total fat and lean mass were not statistically different between groups. However, given that DEXA scans have been demonstrated to consistently underestimate fat mass [28, 30], we considered it relevant to assess body composition by full body MRI as well, especially in a patient population with fat infiltration into atrophic muscle tissue. With full body MRI, differences in total adipose tissue volume nor total lean tissue volume were statistically significant between groups. Still, we consider the observed differences to be clinically relevant, especially since they are in line with previous research [7, 8, 10]. In support, fat ratio was significantly higher in DM1 affected individuals, which seems to be of greater relevance as fat ratio takes loss of lean muscle volume into account. Visceral adipose tissue volume was also significantly higher in DM1 patients, as suggested in literature [7, 10, 31]. The current study is the first to assess body composition in DM1 patients by means of full body MRI, as previous data were based on DEXA or bioelectrical impedance analysis. In the present study we also performed CT to assess upper leg muscle atrophy, demonstrating a difference in CSA of the quadriceps between groups, even though this did not reach statistical significance.

Both 24 h resting EE and corrected resting EE per liter lean tissue were comparable between DM1 affected individuals and matched controls. Even though resting EE of DM1 patients had been researched in three small studies, measurements were based on only short intervals using ventilated hood method [11, 28, 29]. While there seemed to be a difference in resting EE between DM1 patients and healthy matched controls in these other studies, this difference was no longer present after correction for lean muscle volume which is comparable to our data [11, 28, 29]. Since the current study used 24 h whole room calorimetry under standardized circumstances, resting EE estimates can be considered more accurate and precise [32].

Apart from EE under standardized circumstances, DLW was used to asses total EE under free living conditions. While the DLW technique is considered the gold standard for determination of total EE, it had not yet been used in DM1 patients nor in patients diagnosed with other neuromuscular diseases. A study using continuous heart rate registration, with subsequent heart rate VO_2_ calculations, did estimate EE under free living conditions in patients with slowly progressive neuromuscular disease (including DM1) and matched controls [29]. This study described a decreased total EE as a result of a reduced amount of physical activity, in comparison to healthy matched adults, as is in line with our results [29].

The hypothesis of lower total EE in DM1 being the result of relative inactivity, can be confirmed by comparing collected accelerometer data between both groups. Indeed, there was a clear distinction in the amount of physical activity between DM1 affected individuals and healthy adults, with DM1 patients registering less than half the number of steps per day. A previous study measuring physical activity in DM1 patients using accelerometers had similar results, even though data was collected over a shorter seven day period [33]. By extending this period to 15 days in the current study, the effects of possible behavioral adjustments were minimized. Possible causes for a decreased amount of physical activity in DM1 patients do not only consist of muscular impairment, but also participation restrictions in daily and social activities and cognitive impairment [34], even though this was not objectified in the current study.

VO_2_ peak values confirmed a clear distinction in exercise capacity between DM1 affected individuals and healthy controls. When comparing test results to normative values in the Dutch population, DM1 affected individuals’ scores were lower than the lower limit of normal P3 percentile, while healthy individuals’ scores were between P10–P25 [35]. Despite the fact that DM1 patients had a reduced exercise capacity, all of the included participants completed the test until maximal effort based on respiratory exchange ratio [36]. Absolute ventilatory anaerobic threshold and respiratory compensation point values seemed to indicate an early switch to anaerobic metabolism in DM1 patients, yet these differences no longer remained when values were expressed as percentages of the VO_2_ peak. Only one other study has described cardiovascular exercise testing in DM1 patients, even though this was a retrospective evaluation of exercise testing based on clinical indications [37]. Approximately 35% of included DM1 patients was unable to reach maximal effort and the ascertained limited exercise tolerance was presumed to be the result of cardiac and ventilatory deficiencies [37]. In our study, oxygen uptake efficiency slope was also significantly different between groups [38, 39]. The difference in oxygen uptake efficiency slope may indicate pulmonary and/or cardiac involvement with suboptimal O_2_ uptake in the included DM1 participants, yet cardiac and pulmonary data (from regular disease follow-up) was not included in the study.

Oxidative capacity was also evaluated at the level of muscle tissue through CS activity analysis. CS activity is considered a biomarker for muscle metabolic capacity [40, 41]. Even though maximal CS activity was not statistically different between patients and healthy controls, possibly resulting from insufficient statistical power, median values suggest a lower oxidative capacity of muscle tissue of DM1 patients. A lesser oxidative capacity would be secondary to a lower physical activity level as CS activity has been demonstrated to increase after training interventions [42]. Apart from CS, mitochondrial content and function have been demonstrated to increase after aerobic training in DM1 as well [43]. Still, research on DM1 mitochondrial functioning has demonstrated conflicting results [44] while there seems to be an increasing amount of evidence suggesting that mitochondrial dysfunction is part of the DM1 phenotype [12, 44–46]. Interestingly, this did not seem to affect resting EE in our study population.

The main limitation of this study consists of the relatively small sample size. Moreover, we deliberately chose not to collect data on energy intake, which would facilitate the assessment of a potential mismatch in energy intake and expenditure, as the 15-day trial period was already relatively demanding, considering the neuropsychological status of DM1 patients. Also, we did not include cardiopulmonary characteristics and cardiovascular risk profiles, as this would necessitate a large amount of additional analyses in healthy controls. By combining several advanced research techniques in one study, however, we did manage to get a complete overview of metabolic function of DM1 patients, which we were able to compare to healthy, matched controls. Still, results might have been influenced by the fact that six DM1 patients had an impaired glucose tolerance. Finally, thyroid function was not assessed in DM1 affected individuals as part of the study although thyroid function screening is part of standard DM1 patient follow-up. The relationship between thyroid dysfunction and DM1 still remains unclear [2, 47].

The current study did not find a difference in resting EE, but did find a difference in total EE under free living conditions, in oxidative capacity and in the amount of physical activity between both groups. Therefore, overweight in DM1 seems to be the consequence of a structural more positive energy balance. Since CTG repeat expansions of included participants were mostly on the lower end of the scale and only patients with the adult-onset subtype were included in the study, total EE might even be lower in the DM1 population. Moreover, as muscle wasting continues over the course of disease, caloric needs continue to decrease during DM1 patients’ lifetimes. As a result, patient management in DM1 should focus on correcting the caloric mismatch to prevent weight gain, or even on creating a temporary negative energy balance in case of desired weight loss. Ideally, this could be accomplished through a combination of dietary changes and increased physical activity. Due to muscle weakness, fatigue and neuropsychological symptoms (such as lack of motivation and apathy) [48, 49], the ideal type of lifestyle intervention and preferred exercise training modalities remain to be determined. Even though cardiopulmonary fitness was very poor in DM1 affected individuals, patients did manage to complete exercise testing as part of the current study protocol. Moreover, other studies have established the feasibility of long-term exercise interventions and have demonstrated positive effects on patients-reported outcomes, muscle endurance, mitochondrial functioning and functional testing in DM1 [43, 50, 51]. Still, there are insufficient data available to determine an optimal exercise training intervention for DM1 patients at this time [50]. Based on a high level of heterogeneity in DM1 manifestations both on a muscular and neuropsychological level, a personalized intervention seems to be the most suitable. With regards to the lesser muscle mass and low oxygen uptake capacity, exercise interventions should focus both on improving muscle mass and strength as well as increasing endurance performance. Moreover, it would be of added value to determine if weight loss could be beneficial not only on a physical level, but also on the level of social participation and quality of life as these effects have been established in the general population [52, 53].

Apart from a subjective desire to lose weight in many DM1 patients, the data in this study stresses the need for cardiovascular risk management as well. While overweight is known to impose cardiovascular risk on the long-term, specifically visceral obesity has also been linked to insulin resistance, hypertension and atherogenic dyslipidemia [54, 55]. In our study, glucose tolerance was abnormal in 6 out of 15 DM1 patients. Data on the prevalence of subsequent cardiovascular disease in DM1 is scarce, which might be the result of it being considered less important due to reduced survival [56]. As patient management has been improving over the last decade, cardiovascular risk management will become of greater importance.

In conclusion, whole-body EE does not differ in DM1 affected individuals when compared to healthy age-, sex- and BMI-matched controls when assessed under strict dietary and physical activity standardization. However, under free living conditions, daily EE is severely reduced in DM1 patients which can be attributed to a low physical activity level. The low physical activity status, accompanied by a low physical fitness level, represents a key factor responsible for the undesirable changes in body composition in this patient population. In clinical practice, DM1 patient management should include promotion of a more active lifestyle combined with an exercise intervention program to increase physical fitness, increase EE and, as such, prevent overweight and reduce cardiovascular risk.

## Supplementary Material

Supplementary Material
