# Supplementary material for: Energy Expenditure, Body Composition, and Skeletal Muscle Oxidative Capacity in Patients with Myotonic Dystrophy Type 1
Source: J Neuromuscul Dis. 2023 Jul 4;10(4):701–12. doi: 10.3233/JND-230036 (PMC10357167; doi:10.3233/JND-230036)
Supplement: Supplementary Material [file jnd-10-jnd230036-s001.pdf]

Supplementary Table 1

Supplementary Table 1. Body composition determined by DEXA scan			
	DM1 patients (*n*=15)	Healthy controls (*n*=15)	P value
Total lean mass, kg [IQR]	46.74 [43.60-56.16]	48.94 [44.30-60.22]	1.000
Total fat mass, kg	29.42 [24.05-36.63]	23.99 [18.75-28.79]	0.466
